# Antioxidant and Antiaging Activity of *Houttuynia cordata* Thunb. Ethyl Acetate Fraction in *Caenorhabditis elegans*

**DOI:** 10.3390/nu16234168

**Published:** 2024-11-30

**Authors:** Hyeon-Ji Kim, Ji-Su Mun, Suk-Heung Oh, Jun-Hyung Kim

**Affiliations:** 1 Department of Food and Biotechnology, Woosuk University, Wanju 55338, Jeonbuk-do, Republic of Korea; amanda9904@naver.com (H.-J.K.); munjs960208@naver.com (J.-S.M.); shoh@woosuk.ac.kr (S.-H.O.); 2 BIOMAYSIN, Jeongeup 56212, Jeonbuk-do, Republic of Korea; 3 Woosuk Institute of Smart Convergence Life Care (WSCLC), Woosuk University, Wanju 55338, Jeonbuk-do, Republic of Korea

**Keywords:** *Houttuynia cordata*, anti-oxidant, anti-aging, *Caenorhabditis elegans*

## Abstract

Background/Objectives: In aerobic organisms, such as humans, oxygen radicals are inevitably produced. To counteract oxidation, the body generates antioxidant substances that suppress free radicals. However, levels of reactive oxygen species (ROS) increase due to aging and lifestyle factors, leading to exposure to various diseases. While synthetic antioxidants offer advantages like high stability, low cost, and availability, their safety remains controversial. This study aimed to investigate the antioxidant and antiaging activities of *Houttuynia cordata* (HC), which is rich in flavonoids and has excellent antioxidant properties, using *Caenorhabditis elegans* as a model. Methods: Extraction and fractionation of HC were performed to evaluate antioxidant activities (DPPH, ABTS, superoxide radical scavenging activity) and antiaging effects (lifespan). The ethyl acetate fraction (EAF) with the highest activity was selected for further investigation. Results: The EAF of HC exhibited high levels of polyphenols and flavonoids, presenting the highest DPPH, ABTS, and superoxide radical scavenging activities. This fraction increased the activity of antioxidant enzymes in nematodes in a concentration-dependent manner and provided resistance to oxidative stress, reducing ROS accumulation. Additionally, the fraction enhanced the lifespan of nematodes, improved resistance to heat stress, increased survival rates, and decreased the accumulation of aging pigments (lipofuscin). The expression of daf-2, daf-16, and sir-2.1, proteins directly involved in nematode aging, was confirmed. Liquid chromatography/tandem mass spectrometry identified quercitrin in the HC extract, which may contribute to its antioxidant and antiaging effects. Conclusions: The EAF of HC demonstrates significant potential for influencing antioxidant and antiaging, as evidenced by functional investigations using *C. elegans*.

## 1. Introduction

Oxygen is a highly reactive molecule that can readily bind to most elements to form oxides. Aerobic organisms use oxygen from the air to store energy in the form of adenosine triphosphate. This process involves a chain of oxidation reactions that occur within normal metabolism [1]. However, reactive oxygen species (ROS) can be generated during oxygen respiration; chemical substances, air pollution, and metabolic imbalances can convert oxygen to highly reactive ROS, such as hydroxyl radicals, hydrogen peroxide, superoxide, and lipid peroxyl radicals. Appropriate levels of ROS can form a balance with the antioxidant defense system, which reduces ROS levels; however, the ROS concentration in the body increases rapidly due to factors such as aging, frequent exposure to ROS, overeating, stress, drinking, smoking, and irregular lifestyle habits. Being the most predominant cause of aging, increased cellular injury due to ROS accumulation has been discussed as evidence of aging. Aging is the primary risk factor for various conditions, including cardiovascular disease, degenerative disease, and cancer [2]. High ROS concentrations in the body lead to cellular or tissue injury since the powerful oxidative strength of the ROS allows them to oxidize cell membrane lipids and cause protein denaturation and DNA damage. This can lead to diseases such as cellular aging, arteriosclerosis, diabetes, stroke, and cancer [3,4]. It is vital to control imbalances in ROS by consuming antioxidant supplements, which can inhibit the activity of ROS and free radicals in the body.

Synthetic antioxidants overcome the limitations of plant-based natural antioxidants. Most plants exhibit discoloration or rancidification caused by oxidative injury due to heat or light, lipid peroxidation, or prolonged storage, making it difficult to maintain quality over a long period of time. However, while synthetic antioxidants possess advantages such as high stability, low cost, and availability, their safety is still controversial. There is ongoing research on increased carcinogenicity, skin allergies, and gastrointestinal problems, and natural antioxidants are being increasingly explored, which can replace synthetic antioxidants while providing both functionality and safety [5]. Polyphenol compounds, which include flavonoids, catechins, anthocyanins, tannins, isoflavones, lignans, and resveratrol, are predominantly found in plants and are particularly abundant in fruits and leafy vegetables [6]. *Houttuynia cordata* Thunb., belonging to the family *Saururaceae*, is a perennial herbaceous medicinal plant native to China, Japan, and South Korea. *H. cordata* is a traditional medicinal plant with potential diuretic, inotropic, pyrolytic, and antimicrobial effects [7]. The antioxidant [8], anticancer [9], antimicrobial [10], antidiabetic and antithrombotic [11], antiallergic [12], and immune-enhancing [13] effects of *H. cordata* extract are also being explored. In addition, quercetin, the physiologically active substance in *H. cordata*, exhibits various physiological functions [14]. Quercetin is a flavonoid found as a glycoside in various types of fruit, vegetables, and nuts and is known to possess antioxidant, anticancer, anti-inflammatory, antiviral, antiaging, antithrombotic, antiplatelet, and vasodilatory effects [15].

*Caenorhabditis elegans* is an organism with 60% genetic homology to humans; due to its short maximum lifespan, it is a preferred study model in various experiments on extending lifespan and metabolic syndromes [16]. Moreover, as a multicellular organism with a complete genome, *C. elegans* has been used widely to study metabolism and physiological functions in fields such as genetics and developmental biology [17]. In this study, we aimed to analyze the antioxidant effects of *H. cordata* extract and fractions by measuring the 2,2-Diphenyl-1-picrylhydrazyl (DPPH), 2,2′-azino-bis(3-ethylbenzothiazoline-6-sulfonic acid) (ABTS), and superoxide radical scavenging activities. We then collected the ethyl acetate fraction, which showed the most potent antioxidant activity, identified the active components in the fraction, and tested its antioxidant and antiaging potential in a *C. elegans* model.

## 2. Materials and Methods

### 2.1. Extraction and Fractionation

The *H. cordata* powder used in this study was obtained from HANDSHERB (752, Cheonmun-ro, Yeongcheon-si, Gyeongsangbuk-do, Republic of Korea). Extraction was performed by adding 1 kg of *H. cordata* powder to methanol; the mixture was placed in a hot water bath at 50 °C four times for 6 h each. The extract was then concentrated under reduced pressure in a water bath, and 83.73 g of *H. cordata* methanol extract was obtained. The methanol extract was suspended in 1 L of distilled water and then fractionated sequentially in the same quantities of *n*-hexane (21.73 g), methylene chloride (0.73 g), ethyl acetate (11.37 g), and *n*-butanol (12.54 g), to obtain different fractions. The extract and fractions required for the experiment were sealed and stored in the dark until use.

### 2.2. Evaluation of Polyphenol and Flavonoid Contents

The polyphenol and flavonoid contents were determined as described previously [18,19]. The Folin–Ciocalteau colorimetric method was used to measure the polyphenol content of the *H. cordata* methanol extract and fractions. To prepare the samples, 10 mL of ethanol was added to 20 mg of each specimen and diluted to different concentrations. After adding 100 μL of Folin–Ciocalteu reagent to 100 μL of sample, the mixture was homogenized for 2 min. Next, 800 μL of 0.1 M sodium carbonate was added, and the mixture was reacted by placing it in a 40 °C heating bath for 20 min. The samples were then frozen, and a microplate reader was used to measure absorbance at 700 nm. Finally, to calculate the total polyphenol content, we used a tannic acid standard to derive a first-order calibration equation by concentration (y = 0.0006x + 0.0351 R^2^ = 0.9975) and expressed the polyphenol content in terms of mg tannic acid equivalent (TAE)/g. To measure the flavonoid content in the *H. cordata* methanol extract and fractions, we first pretreated the samples using the same methods as those for polyphenol measurement. After adding 30 μL of 5% NaNO_2_ to 1 mL of sample, the mixture was reacted for 5 min at 20 °C. Then, 30 μL of 10% AlCl_3_ and 200 μL of 1 M NaOH were mixed, and a microplate reader was used to measure the absorbance at 510 nm. Finally, to calculate the flavonoid content, we used a quercetin standard to derive a first-order calibration equation by concentration (y = 0.0009x + 0.0322 R^2^ = 0.9955) and expressed the total flavonoid content in terms of mg quercetin equivalent (QE)/g.

### 2.3. Measurement of DPPH and ABTS Radical Scavenging Activities

The radical scavenging activities were determined, as described previously [20,21]. We measured the reducing activity of the *H. cordata* methanol extract and fractions based on the electron-donating ability of DPPH. First, the *H. cordata* methanol extract and the fractions were adjusted to varying concentrations in ethanol; 50 μL of each sample was added to a 96-well plate, and 200 μL of 0.2 mM DPPH solution (EtOH) was added to each well. The plate was reacted in a dark room at room temperature for 30 min, and the absorbance was then measured at 517 nm. L-ascorbic acid was used for the positive control group, and the antioxidant activity was estimated graphically by comparison with the absorbance of a negative control group with no added sample. The activity of each sample was measured in triplicate. To measure the ABTS radical scavenging activity of the *H. cordata* methanol extract and fractions, equal quantities of 7.4 mM ABTS and 2.6 mM K_2_S_2_O_8_ were mixed; the mixture was left in a dark room at room temperature for 24 h to induce radical formation. The ABTS solution was then diluted until the absorbance value reached 0.7 ± 0.03. After adding 100 μL of ABTS solution to 10 μL of the test samples at different concentrations, the mixtures were reacted for 10 min in a dark room at room temperature, followed by measuring the absorbance at 732 nm. L-ascorbic acid was used for the positive control group, and the antioxidant activity was estimated graphically by comparison with the absorbance of a negative control group with no added sample. The activity of each sample was measured in triplicate.

### 2.4. Assessment of Superoxide Radical Scavenging Activity

The superoxide radical scavenging activity was determined as described previously [22,23]. To measure the ability to scavenge superoxide radicals produced during the enzymatic activity of xanthine oxidase, we mixed 10 μL samples of *H. cordata* methanol extract and fractions prepared to different concentrations (250, 500 μg/mL) with phosphate buffer (pH 7.8) containing 100 μM nitro blue tetrazolium (NBT) and 250 μM xanthine, and incubated the mixture for 5 min. We then added 100 μL of xanthine oxidase (0.05 U/mL) and incubated the mixture at 37 °C for exactly 20 min, after which 69 mM superoxide dismutase (SOD) was added to arrest the reaction. Finally, the absorbance was measured at 570 nm. The activity of each sample was measured in triplicate.

### 2.5. Caenorhabditis Elegans Culture

*C. elegans* was obtained from the Caenorhabditis Genetics Center (CGC; University of Minnesota, Minneapolis, MN, USA). We used wild type N2, and the transgenic strains CF1553 muls84[pAD76(SOD-3 p::GFP + rol-6(su1006)], CL2070dvls70[HSP-16.2::GFP+rol-6 (su1006)], and TJ356 zIs356 [daf-16p::daf-16a/b::GFP + rol-6(su1006)]. *C. elegans* was cultured at 20 °C on a Nematode Growth Medium (NGM) agar plate with *Escherichia coli* (OP50). To collect the eggs, a bleaching solution (NaClO, 5 M KOH) was applied to nematodes washed with M9 buffer [24]. The samples used in the experiments were applied to sterile NGM plates in the form of stock solutions in dimethyl sulfoxide (DMSO), and the final DMSO concentration in all conditions was maintained at 0.1% (*v*/*v*).

### 2.6. Measurement of Antioxidant Enzyme Activity in C. elegans

The ethyl acetate fraction of *H. cordata* at varying concentrations (250, 500 μg/mL) was added to the plate, and synchronized nematodes were cultured to day 2 adults, when they were washed three times with M9 buffer, homogenized, and used to measure antioxidant enzyme activity (homogenization buffer: 10 mM Tris-HCl, 150 mM NaCl, 0.1 mM EDTA, pH 7.5). To measure SOD activity, a reaction mixture (1.6 mM xanthine and 0.48 mM NBT) prepared in 10 mM phosphate buffer (pH 8.0) was mixed with 10 μL of the sample at different concentrations and incubated for 5 min at room temperature. We then added 100 μL of xanthine oxidase (0.05 U/mL) and incubated the mixture at 37 °C for 20 min before adding 69 mM sodium dodecyl sulfate (SDS) to arrest the reaction. Finally, the absorbance was measured at 570 nm [25]. To measure catalase activity, 25 mM H_2_O_2_ was reacted with 50 μL of test samples at different concentrations, and the absorbance was measured at 240 nm for 4 min at 20 s intervals [26].

### 2.7. Evaluation of Ros Accumulation in C. elegans

To analyze ROS in *C. elegans* cells, we used synchronized day 4 adults cultured on plates treated with *H. cordata* ethyl acetate fraction. Reaction with 2′,7′-dichlorodihydro fluorescein diacetate (H_2_DCF-DA) was used for fluorimetry analysis. Four-day adults were exposed to M9 buffer containing 100 μM juglone for precisely 2 h, then moved to a 96-well plate containing M9 buffer. Next, 50 μM H_2_DCF-DA was added, and the fluorescence intensity was measured for 2 h at 30 min intervals at an excitation wavelength of 485 nm and an emission wavelength of 535 nm [27].

### 2.8. Stress Tolerance Assessment

To investigate the effects of *H. cordata* ethyl acetate fraction on oxidative stress in *C. elegans*, we cultured synchronized nematodes on plates treated with *H. cordata* ethyl acetate fraction. Seven-day-old nematodes were moved to a 96-well plate containing M9 buffer with 1 mM juglone, and nematode survival was checked every hour. Specifically, the nematodes were touched with a platinum wire, and non-responsive ones were considered to be dead [25]. To investigate the effects of *H. cordata* ethyl acetate fraction on heat stress resistance in *C. elegans*, which has an optimal growth temperature of 20 °C, we cultured synchronized nematodes on NGM plates treated with *H. cordata* ethyl acetate fraction (250, 500 μg/mL). Four-day adult nematodes were moved to a fresh NGM plate, heat stress was applied by culturing at a high temperature of 36 °C, and the hourly survival rates were recorded [28].

### 2.9. Fluorescent Expression of sod-3, daf-16, and hsp16.2 in C. elegans

The GFP-fused transgenic *C. elegans* strain CF1553 was cultured on a plate treated with *H. cordata* samples at different concentrations. The expression of GFP in 3-day adults was examined after anesthesia using 4% sodium azide [29].

We used the transgenic *C. elegans* strain TJ356, which expresses DAF-16::GFP, to enable the nuclear localization of DAF-16 to be examined. We cultured adult nematodes for 3 days and then washed them with M9 buffer for use in experiments [30].

The *C. elegans* strain CL2070, which contains the transgene HSP-16.2::GFP, was cultured under the same conditions to 3-day adults and used to test the mitigation of heat shock stress. Adult nematodes were incubated for 2 h at 36 °C and recovered for 2 h at 20 °C, following which GFP expression was examined using a fluorescence stereomicroscope (OLYMPUS SZX16, Shinjuku Ward, Tokyo, Japan) [31]. To evaluate the accumulation of lipofuscin in *C. elegans*, synchronized nematodes were cultured on a medium treated with samples of different concentrations. Eight-day adults were anesthetized using 4% sodium azide, and a fluorescence stereomicroscope was used to examine the expression of lipofuscin. For quantification and analysis of fluorescence intensity, images were captured using an OLYMPUS SZX16 (Japan) and analyzed using the Image J software 1.54K version [32].

### 2.10. Evaluating the Longevity Effects and Analyzing Protein Expression

To investigate the effects of *H. cordata* extract and fractions on *C. elegans* longevity, we added a bleaching solution and isolated the eggs. The eggs were then cultured (20 °C) on an NGM plate with an aqueous solution of *H. cordata* to synchronize the growth stage, and the survival rates were determined. For accurate measurements and to prevent contamination of the NGM plate, we moved the nematodes to a new plate once per day until Day 7, after which they were moved to a new NGM plate every 2 days. To check survival, the nematodes were touched with a platinum wire, and the non-responsive ones were considered to be dead [33]. To investigate the effects of *H. cordata* ethyl acetate fraction on antiaging proteins in *C. elegans*, synchronized nematodes were cultured in a medium treated with different concentrations of the fraction. Two-day adults were collected and washed using M9 buffer (0.5% NaCl, 0.6% Na_2_HPO_4_, 0.3% KH_2_PO_4_, 0.1% NH_4_Cl). A homogenization buffer was then added; the homogenized nematodes were centrifuged, and a bicinchoninic acid assay was performed. The protein was mixed with the same quantity of sample loading buffer and boiled for 5 min at 95 °C. SDS-polyacrylamide gel electrophoresis was performed, and the protein was transferred (overnight) to a 0.2 µm polyvinylidene fluoride membrane. The membrane was blocked by soaking in 5% bovine serum albumin for 3 h [34]. SIR-2.1 (PA1-16933), DAF-2 (MBS418360), and secondary antibody (Goat Anti-Rabbit IgG H&L, ab205718) were used to quantify changes in protein content.

### 2.11. Liquid Chromatography-Tandem Mass Spectrometry Analysis

To identify the key compounds in *H. cordata* extract and the ethyl acetate fraction, we used ultra-performance liquid chromatography (LC; Acquity system, Waters, Milford, CT, USA) with tandem mass spectrometry (MS-MS; Xevo TQ-X triple quadrupole mass spectrometer [Waters, Milford, CT, USA]) using electrospray ionization. Using a hybrid reverse-phase column (Synergi Hydro-RP, 4 μm; Phenomenex, Torrance, CA, USA) maintained at 30 °C, we performed analysis for 10 min at a flow rate of 0.2 mL/min. For analysis, we used multiple reaction monitoring with a pressure of 7 bar, source temperature of 150 °C, desolvation temperature of 350 °C, cone gas flow of 150 L/h, desolvation gas flow of 600 L/h, and collision gas flow of 0.15 mL/min. For the mobile phase solvents A and B, 0.1% formic acid and 0.1% formic acid were used in acetonitrile, respectively. The standards were purchased from Sigma Aldrich. *H. cordata* extract and ethyl acetate fraction were diluted and dissolved to a concentration of 100 ppm and then centrifuged for 10 min at 13,000× *g*. The supernatant was collected and diluted to different concentrations to make the test samples.

### 2.12. Statistical Analyses

Statistical data are presented in terms of the mean value and the standard error of the mean (mean ± S.E.M.), and Student’s *t*-test was used to analyze statistically significant differences between groups. For the *C. elegans* survival analysis, we used the log-rank test method, and *p* values of * *p* < 0.05, ** *p* < 0.01, and *** *p* < 0.001 were considered significant, strongly significant, and very strongly significant, respectively.

## 3. Results and Discussion

### 3.1. Measurement of Polyphenol and Flavonoid Contents

Phenolic compounds are secondary metabolites produced by plants and are prevalent throughout the plant kingdom. These include all molecules with an aromatic ring and at least one hydroxyl (-OH) group. Phenolic compounds that can be obtained from plants include phenolic acid, flavonoids, and tannins; polyphenols are the most readily accessible antioxidants [6]. Research has been conducted on the effects of the dietary intake of phenolic compounds on the cardiovascular system [6], neurodegenerative disease [35], and cancer [36]. We measured the polyphenol and flavonoid content in *H. cordata* methanol extract and various solvent fractions. The ethyl acetate fraction showed the highest polyphenol content, at 881.3 µg TAE/mL. Song. et al. have reported an excellent polyphenol content of 359.3 μg/mL for the ethyl acetate fraction of *H. cordata* [37], while Moon, et al. have reported a polyphenol content of more than 1800 mg/100 g for an 80% methanol extract of *H. cordata* [38]. The experimental methods used in this study are identical to those reported in the two previous papers. However, differences in the sample extraction methods, cultivation regions, and cultivation periods are believed to have resulted in variations in the measured total polyphenol content.

Correspondingly, the ethyl acetate fraction showed a very high flavonoid content, at 1941.1 μg QE/mL (Table 1). Kim. et al. reported a flavonoid content of 1.40 ± 1.80 mg/g for *H. cordata* [4], whereas Moon. et al. reported a flavonoid content of 667.3 ± 13.3 mg/100 g for an 80% methanol extract of *H. cordata* [38]. Considering experimental method variations, as well as differences in *H. cordata* cultivation time and extraction conditions, the exact values are likely to vary, but the above results consistently demonstrate that *H. cordata* contains high levels of polyphenols and flavonoids. The total flavonoid content was also measured using the same method in all cases. However, differences in the sample extraction methods, cultivation regions, and cultivation periods are thought to have caused the observed variations.

### 3.2. Measurement of DPPH and ABTS Radical Scavenging Activity

**Figure 1:**
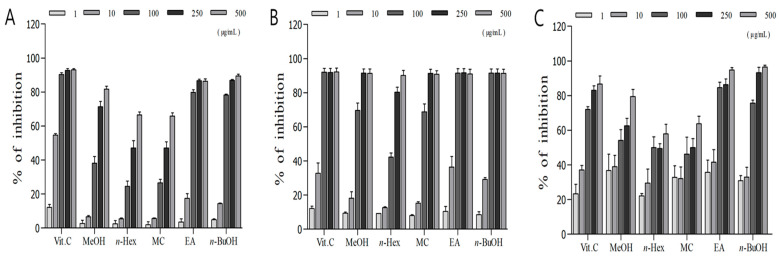
(**A**) DPPH free radical scavenging effects of the methanol extract and its fractions from *Houttuynia cordata* Thunb. (**B**) ABTS radical scavenging effects of the methanol extract and its fractions from *Houttuynia cordata* Thunb. (**C**) Xanthine-originated superoxide quenching activities of the methanol extract and its fractions of *Houttuynia cordata* Thunb. Vitamin C was used as a positive control for experiments A, B, and C. Vit. C, vitamin C; MeOH, methyl alcohol; *n*-Hex, *n*-Hexane; MC, methylene chloride; EA, ethyl acetate; *n*-BuOH, *n*-Butanol.

The DPPH assay, which is based on the principle that DPPH solution is decolorized in interaction with antioxidants, has been widely used to study the development of antioxidants from natural substances [10]. We measured the DPPH radical scavenging activity of *H. cordata* methanol extract and various solvent fractions. As shown in Figure 1A, the DPPH radical scavenging activity of the ethyl acetate fraction increased dose-dependently and was superior to that of the other fractions (ethyl acetate fraction IC_50_ value, 103.3 μg/mL). Yun. et al. have reported excellent DPPH free radical scavenging activity (FSC_50_) of 12.0 μg/mL on reacting *H. cordata* ethyl acetate fraction with DPPH solution in a 1:1 ratio [39]. Cho and Kwon have also reported excellent DPPH radical scavenging activity, with an IC_50_ of 27.9 μg/mL when *H. cordata* ethanol extract is reacted with DPPH solution in a 1:1 ratio [40]. When comparing this experiment with the two previous ones, the mixing ratio of the sample to the DPPH reagent in this study was 1:5, differing in the sample addition ratio. This is likely to contribute to differences in the results. Additionally, variations in the cultivation period, conditions, and regions of *Houttuynia cordata* could lead to differences in the plant’s constituent content, which may, in turn, affect its radical scavenging activity. In addition, ABTS radicals are also widely used to measure the antioxidant activity of hydrophilic and hydrophobic compounds while minimizing the effects on pigments in the samples [41]. The ABTS radical scavenging activity of *H. cordata* methanol extract and various solvent fractions revealed that the ethyl acetate fraction showed a dose-dependent increase in scavenging activity with an IC_50_ of 13.83 μg/mL, demonstrating better ABTS radical scavenging activity than that of the other fractions and the L-ascorbic acid control group (Figure 1B). These findings are consistent with those of a previous study by Park, et al. [42], wherein the ethyl acetate fractions of *H. cordata* showed ABTS radical scavenging activity superior to that of the other fractions. Similarly, Lee. et al. reported an ABTS radical scavenging activity of 85.7% for an ethyl acetate fraction of *H. cordata*, which was the highest when compared to that for other fractions [10]. In this study, when expressing radical scavenging activity as a percentage, it was confirmed that the sample group with 100 μg/mL addition exhibited a scavenging activity of 91.5%.

### 3.3. Measurement of Superoxide Radical Scavenging Activity

Xanthine oxidase is an enzyme found in many species, including humans. It produces superoxide anions and hydrogen peroxide from oxygen [43], increases cytotoxicity, and is involved in aging, diabetes, and several neurodegenerative diseases, including dementia [11]. Therefore, inhibiting xanthine oxidase activity may have potential antioxidant effects, mitigating oxidative damage by reducing the production of free radicals [44]. We measured the superoxide radical scavenging activity of *H. cordata* methanol extract and various solvent fractions. As shown in Figure 1C, the ethyl acetate fraction showed a dose-dependent increase and the highest scavenging activity (IC_50_ value, 5.2 μg/mL), which was higher than that of the L-ascorbic acid control group (IC_50_ value, 12.4 μg/mL). This is in contrast to a previous report by Bae [45], wherein they measured the ability of patchouli extract to scavenge superoxide radicals produced by the enzymatic action of xanthine/xanthine oxidase. IC_50_ value, 118.0 μg/mL), our results demonstrated remarkable superoxide radical scavenging activity of the *H. cordata* ethyl acetate fraction. Jang, et al. also measured the superoxide radical scavenging activity of ethyl acetate fractions of a chestnut tree native to Jeju (IC_50_ value, 16.0 μg/mL), chrysanthemum (IC_50_ value, 17.0 μg/mL), Korean raspberry (IC_50_ value, 57.0 μg/mL), and evening primrose (IC_50_ value, 74.0 μg/mL) [46]. Comparative analysis revealed that the superoxide radical scavenging activity of *H. cordata* ethyl acetate fraction was remarkably higher than those of all these extracts. Thus, the *H. cordata* ethyl acetate fraction exhibits promising potential as an exceptional antioxidant.

### 3.4. Measurement of Antioxidant Enzyme (SOD, Catalase) Activity in C. elegans

**Figure 2:**
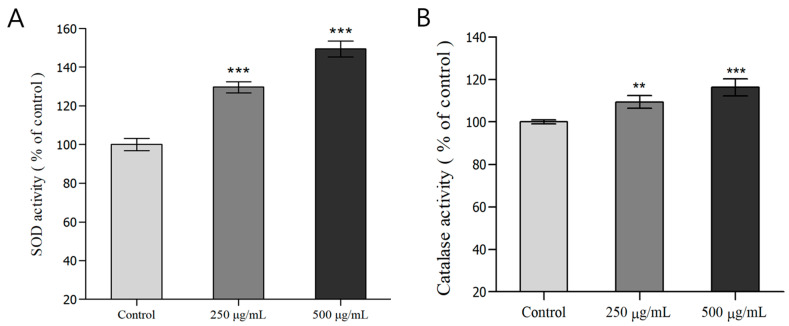

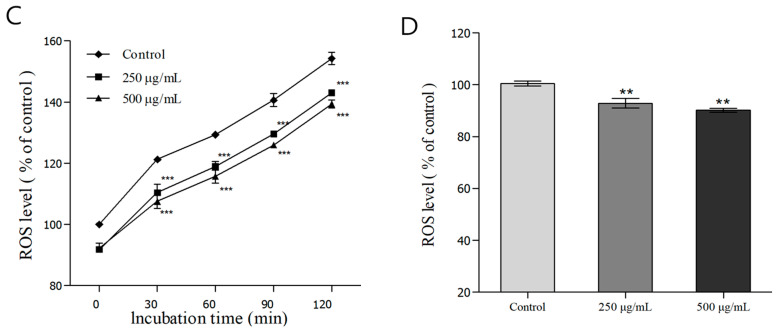
Effects of antioxidant enzyme activities and analysis of intracellular reactive oxygen species (ROS) accumulation of wild-type N2 nematode from *Houttuynia cordata* Thunb. (**A**) Superoxide dismutase (SOD) activity is expressed as the percentage of the scavenging amount of control. (**B**) Catalase activity was calculated from the concentration of residual H_2_O_2_ measured using spectrophotometry. (**C**) The worms were incubated with 100 μM juglone for 2 h and subsequently treated with the fluorescent probe H_2_DCF-DA. Intracellular ROS accumulation was quantified spectrophotometrically at an excitation wavelength of 485 nm and an emission wavelength of 535 nm. The plates were read from 0 min to 120 min. (**D**) Average percentages of intracellular ROS levels. Differences compared with the control group were considered significant at ** *p* < 0.01, *** *p* < 0.001 using one-way analysis of variance (ANOVA).

Using *C. elegans* treated with *H. cordata* ethyl acetate fraction, we measured the SOD and catalase activity in the body. SOD converts superoxide (O_2_^−^) to H_2_O_2_ and oxygen molecules via cyclic oxidative reactions, including introducing metals (Mn, Zn, Cu, Fe) to the active sites, thereby removing ROS. Catalase converts H_2_O_2_ into oxygen and water, eliminating radicals in cells and thereby protecting against oxidative stress [47,48,49]. Analysis of SOD activity in *C. elegans* treated with the ethyl acetate fraction of *H. cordata* revealed that the groups treated with 250 and 500 μg/mL *H. cordata* ethyl acetate fractions showed a 29.6% and 49.4% increase in SOD activity (*** *p* < 0.001), respectively, relative to that of the control group. In addition, the groups treated with 250 and 500 μg/mL *H. cordata* ethyl acetate fractions showed a 9.4% (** *p* < 0.005) and 16.2% (*** *p* < 0.001) increase in catalase activity, respectively, relative to that of the control group. Thus, the *H. cordata* ethyl acetate fraction increases both SOD and catalase activity in a dose-dependent manner (Figure 2A,B). According to a report by Kwon, et al. [50], treatment of liver tissue with a mixed extract of 90 g *H. cordata*, 2 g *Schisandra chinensis*, 2 g *Lycium chinense*, 4 g *Torilis japonica*, and 2 g *Epimedium koreanum* exhibits strong SOD and catalase activity. Meanwhile, in a report by Ha [51], in mice with 2,3,7,8-tetrachlorodibenzodioxin-induced hepatotoxicity, *H. cordata* administration increased SOD and catalase activity by 46% and 50%, respectively.

Liliang Ju et al. also observed a remarkable dose-dependent increase in SOD activity when C_2_C1_2_ cells were treated with 25, 50, or 100 μg/mL *H. cordata* extract [52]. In summary, the ethyl acetate fraction of *H. cordata* significantly increased SOD and catalase activity in *C. elegans*, helping defense mechanisms against injuries due to oxidative stress.

### 3.5. Analysis of ROS in C. elegans

DCF-DA is a fluorescent imaging dye that enters via the cell membrane and is hydrolyzed enzymatically by intracellular esterase, resulting in non-fluorescent DCF-H. Principally, DCF-H then reacts with intracellular ROS and is oxidized to DCF, which is strongly fluorescent [53]. ROS reduce the activity of antioxidant enzymes in the body and are known to cause various diseases by damaging cells [54]. To investigate the ability to reduce ROS accumulation in *C. elegans* cells, we measured fluorescence due to the reaction between H_2_DCF-DA reaction and intracellular ROS in *C. elegans* treated with different concentrations of *H. cordata* ethyl acetate fraction (250, 500 μg/mL). The decrease in fluorescence over 120 min due to ROS was 6.5% and 9.1% in the groups treated with 250 and 500 μg/mL ethyl acetate fractions, respectively, which was a significant decrease compared to the control (Figure 2C). The decrease in fluorescence at 120 min was 7.2% and 10.9% in the groups treated with the 250 and 500 μg/mL ethyl acetate fractions, respectively, showing a significant decrease compared to the control. Therefore, the *H. cordata* ethyl acetate fraction effectively inhibited the accumulation of ROS in *C. elegans* cells (Figure 2C,D). Yun, et al. reported that, in HaCaT cells with UVB-induced ROS generation, the *H. cordata* ethyl acetate fraction showed better ROS scavenging activity than that of the *H. cordata* ethanol extract [10]. Meanwhile, Lim. et al. reported a significant and dose-dependent decrease in ROS after treating RAW 264.7 cells with *H. cordata* extract and lipopolysaccharide, demonstrating the antioxidant potential of *H. cordata* extract [8]. In summary, the ethyl acetate fraction of *H. cordata* significantly mitigated the accumulation of ROS in *C. elegans*, suggesting that it could help prevent cell injury and inflammation.

### 3.6. Evaluating Oxidative and Heat Stress Resistance

**Figure 3:**
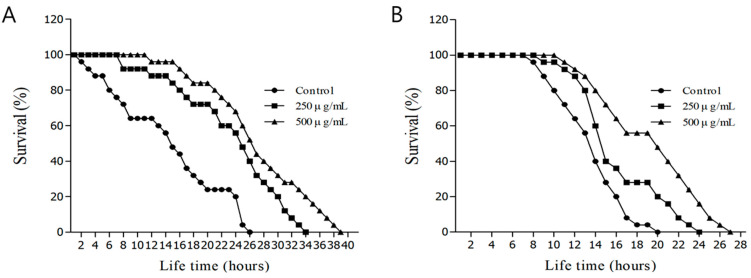
(**A**) Effects of ethyl acetate fraction of the *Houttuynia cordata* Thunb. on the stress tolerance of wild-type N2 nematodes. For the oxidative stress assays, worms were transferred to a 96-well plate containing 1 mM of juglone liquid culture, and then their viability was scored. (**B**) Effects of ethyl acetate-soluble fraction from *Houttuynia cordata* Thunb on the thermal stress tolerance of wild-type N2 nematodes. To assess thermal tolerance, worms were incubated at 36 °C, and their viability was scored. Statistical difference between the curves was analyzed using a log-rank test.

Juglone is a substance produced by walnut trees that is known to induce oxidative stress, cell membrane injury, necrosis, and apoptosis [55,56]. We confirmed that an aqueous solution of *H. cordata* ethyl acetate fraction affected *C. elegans* survival under conditions of oxidative stress. The maximum survival time in the control group after induction of oxidative stress by exposure to M9 buffer containing 1 mM juglone was only 26 h. Meanwhile, the maximum survival times in the groups treated with 250 μg/mL and 500 μg/mL *H. cordata* ethyl acetate fractions were 34 and 39 h, respectively. The mean survival time in the control group after induction of oxidative stress was 13.5 ± 1.0 h, whereas the mean survival times in the groups treated with 250 μg/mL and 500 μg/mL *H. cordata* ethyl acetate fraction were 23.3 ± 1.1 h and 26.8 ± 1.2 h, respectively, representing improvements of 43.1% and 57.0%, relative to that of the control (Figure 3A and Table 2). Heat stress leads to organ dysfunction and increased mortality; the lifespan of *C. elegans* is closely related to heat stress [28]. When we investigated the effects of *H. cordata* ethyl acetate fraction on heat stress resistance in *C. elegans*, the mean survival time in the control group at 36 °C was 14.3 ± 0.4 h, and the maximum survival time was 20 h. Meanwhile, in the group treated with 250 μg/mL *H. cordata* ethyl acetate fraction, the mean survival time was 17.2 ± 0.5 h, and the maximum survival time was 24 h, whereas in the group treated with 500 μg/mL *H. cordata* ethyl acetate fraction the mean survival time was 18.9 ± 0.5 h and the maximum survival time was 27 h, suggesting an increase in heat stress resistance by up to 32.5% (Figure 3B and Table 2). The optimal growth temperature for *C. elegans* is 20 °C; on exposure to a high temperature of 36 °C, its movements become sluggish, the nematodes stop feeding, and they rapidly die [57]. In summary, the ethyl acetate fraction of *H. cordata* was effective in increasing resistance to oxidative and heat stress, thus extending the lifespan of *C. elegans*.

### 3.7. Evaluation of Fluorescence Expression and Accumulation in C. elegans

**Figure 4:**
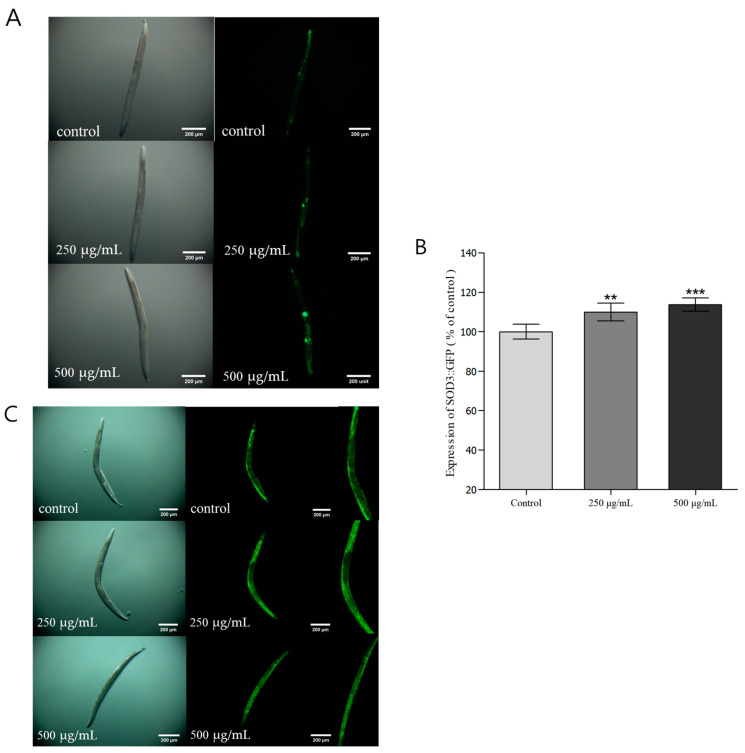
The effect of the ethyl acetate fraction from *Houttuynia cordata* Thunb. on the expression of SOD-3::GFP (CF1553) and DAF-16::GFP (TJ356) was determined using transgenic nematodes. (**A**) Images of SOD-3::GFP expression of CF1553 nematodes in the presence or absence of the ethyl acetate fraction of *Houttuynia cordata* Thunb. (**B**) The mean GFP-expressing intensity of CF1553 mutants was expressed as the mean S.E.M. of values from 100 worms (**A**). (**C**) Images of DAF-16::GFP expression of TJ356 nematodes in the presence or absence of the ethyl acetate fraction of *Houttuynia cordata* Thunb. Data are expressed as the mean ± standard deviation of three independent experiments (N = 3). Differences compared with the control were considered significant at ** *p* < 0.01 and *** *p* < 0.001 using one-way ANOVA.

**Figure 5:**
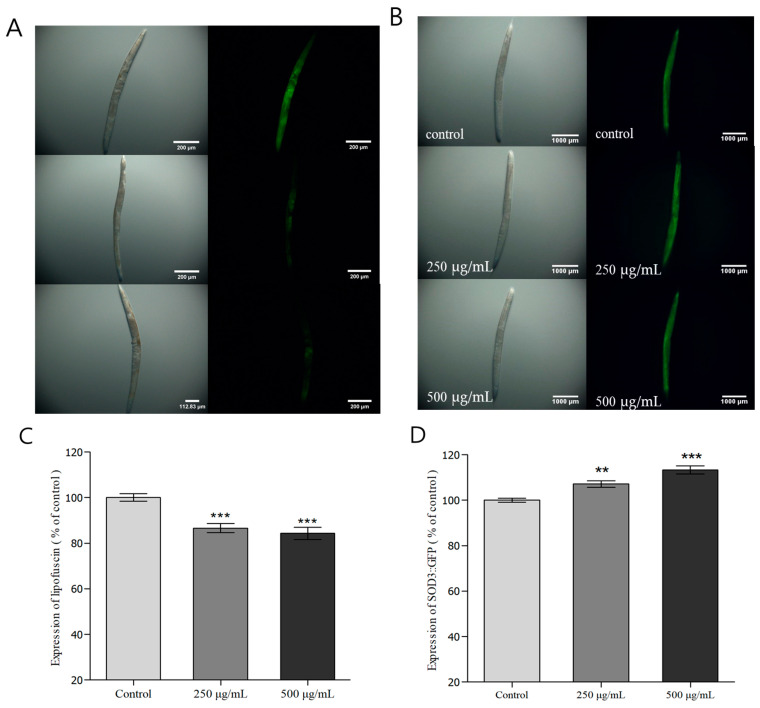
Effect of ethyl acetate fractionation on lipofuscin and HSP-16.2::GFP accumulation in *Houttuynia cordata* thumb. The effect of ethyl acetate fractionation of *Houttuynia cordata* on lipofuscin expression was measured using wild-type N2 (**A**,**C**), and HSP-16.2::GFP was measured using the transgenic nematode CL2070 (**B**,**D**). Images of expression of worms in the presence or absence of ethyl acetate fraction. The mean fluorescence intensity of the mutant was represented as mean ± S.E.M. of values from the 80 worms experiment. The fluorescence intensity was quantified using the Image software by determining the average pixel intensity. Data are expressed as the mean ± standard deviation of three independent experiments (N = 3). Differences compared with the control were considered significant at ** *p* < 0.01 and *** *p* < 0.001 using one-way ANOVA.

SOD-3 is a type of SOD present in mitochondria, forming ionic bonds with Mn, and protects the organism by inhibiting protein denaturation and oxidative damage. As an antioxidant enzyme in the transgenic *C. elegans* strain CF1553, SOD-3 is controlled by the insulin/IGF1 signaling pathways (IIS), and the expression and regulation of SOD-3 are associated with stress resistance and longevity [58,59]. Therefore, in this study, we investigated the expression of SOD-3::GFP in *C. elegans* treated with *H. cordata* ethyl acetate fraction. The groups treated with 250 and 500 μg/mL *H. cordata* ethyl acetate fractions showed 10.0% and 13.7% increases in SOD-3::GFP expression relative to that of the control group (Figure 4A,B). Thus, the ethyl acetate fraction of *H. cordata* increased SOD-3::GFP expression in the transgenic CF1553 strain of *C. elegans* in a dose-dependent manner and was thus determined to increase resistance to oxidative stress. DAF-16 is a protein associated with stress resistance, formation of the dauer stage, and lifespan in *C. elegans*. It is a core protein in the IIS that is transported from the cytoplasm to the nucleus and inhibited by the DAF-2 receptor in the same pathway [60]. The TJ356 strain of *C. elegans* used in this experiment contained a DAF-16::GFP transgene, which allows us to inspect changes in DAF-16 localization. We conducted an experiment to investigate the extent to which *H. cordata* ethyl acetate fraction affects the localization of DAF-16 protein to the nucleus, as well as the extension of lifespan by DAF-16. There was a significant increase in the movement of DAF-16 to the nucleus in groups treated with different concentrations of the ethyl acetate fraction, suggesting that the ethyl acetate fraction of *H. cordata* could suppress aging in *C. elegans* (Figure 4C). Heat-shock proteins (HSPs) protect cells by degrading toxic protein aggregates and acting as a chaperone to ensure the degradation of misfolded proteins [61]. HSP-16.2 is a low molecular weight (15–30 KDa) polypeptide in the class of small HSPs that are found in most eukaryotic organisms. One of the genetic responses produced by *C. elegans* subjected to heat shock is expressing a gene that codes for 16 KDa heat shock proteins (hsp16s) [62]. HSP-16 expression can be considered a measure of *C. elegans* ability to respond to stress, and HSP-12, particularly, is activated due to stress when *C. elegans* is exposed to high temperatures [63]. The CL2070 model is useful for measuring HSP-16.2::GFP expression by inducing widespread GFP expression throughout the body following heat shock. We determined the expression of HSP-16.2::GFP and observed that the groups treated with 250 and 500 μg/mL *H. cordata* ethyl acetate fraction showed 7.1% and 13.3% increase in HSP-16.2::GFP expression, respectively, relative to that of the control group (Figure 5B,D). In summary, when *C. elegans* was exposed to a high temperature, the ethyl acetate fraction of *H. cordata* was found to increase resistance to heat stress in a dose-dependent manner. Lipofuscin is used as a marker to determine the rate of aging and overall health of *C. elegans* since it is expressed in *C. elegans* organs, is autofluorescent, and accumulates in cells with aging [64]. The groups treated with 250 and 500 μg/mL *H. cordata* ethyl acetate fraction showed a 13.5% and 15.7% reduction in lipofuscin accumulation relative to that in the control group, demonstrating that the ethyl acetate fraction of *H. cordata* was effective at reducing lipofuscin accumulation in *C. elegans* (Figure 5A,C).

### 3.8. Evaluation of Lifespan Extension and Protein Expression

**Figure 6:**
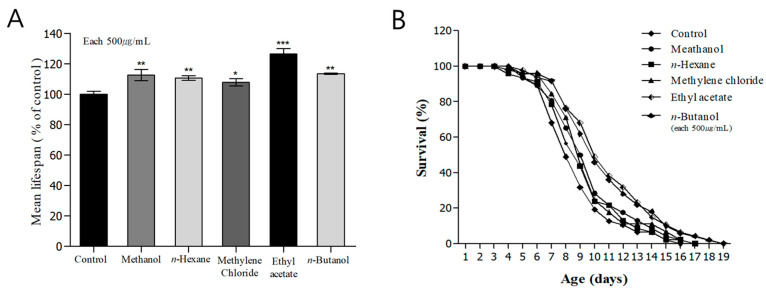

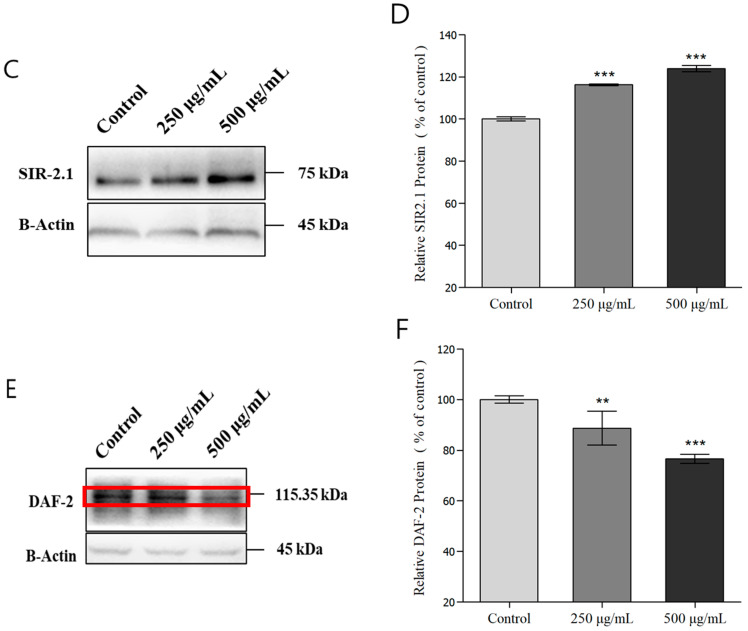
Effects of fractions from *Houttuynia cordata* Thunb on the lifespan and western blotting of wild-type N2 nematodes. Worms were grown in the Nematode Growth Medium agar plate at 20 °C in the absence or presence of fractions. The number of worms used per each lifespan assay experiment was 50, and three independent experiments were repeated (N = 3). (**A**) The mean lifespan of the N2 worms was calculated from the survival curves (**B**). (**B**) The mortality of each group was determined by daily counting of live and dead animals. Statistical difference between the curves was analyzed using a log-rank test. Error bars represent the S.E.M. Differences compared to the control were considered significant *** *p* < 0.001 using one-way ANOVA. (**C**) The increase in SIR-2.1 protein expression was confirmed using immunoblotting of the proteins of N2 nematodes grown in a medium supplemented with different concentrations of *Houttuynia cordata* Thunb ethyl acetate fraction. (**D**,**F**) The immunoblotting results of each protein expression in N2 nematodes were quantified and analyzed by fusion 2.0 and graphed. (**E**) Changes in DAF-2 protein content of nematodes grown under the same conditions as (**C**). The band inside the red box is the daf-2 protein expression. Differences compared with the control were considered significant at * *p* < 0.05, ** *p* < 0.01, and *** *p* < 0.001 using one-way ANOVA.

In addition to genetic factors, environmental factors, including temperature and oxidative conditions, also play an important role in regulating the lifespan of *C. elegans*. Genetic and environmental factors have been reported to affect the rate of aging [65]. Therefore, in this study, we aimed to investigate the effects of *H. cordata* extract and fractions on the lifespan of *C. elegans*. The mean lifespan of the control group was 8.9 ± 0.2 h, and the maximum survival time was 16 days. In the groups treated with *H. cordata* methanol extract, excluding the *n*-hexane fraction, methylene chloride fraction, *n*-butanol fraction, and ethyl acetate fractions, the mean lifespans of *C. elegans* were 9.6 ± 0.2, 9.6 ± 0.2, 9.9 ± 0.3, and 10.9 ± 0.3 h, respectively, whereas the maximum survival times were 17, 17, 19, and 19 days, respectively, representing a 22.1% increase in *C. elegans* life-extending effects using *H. cordata* ethyl acetate fraction, compared to that of the control group (Figure 6A,B and Table 3). *C. elegans* is the first model organism in which long-life mutations have been discovered in vivo and studied with regard to aging, and there are signaling pathways and molecular mechanisms that regulate the lifespan. The IIS is one of the representative pathways regulating aging, including major proteins in the IIS, such as DAF-2, DAF-16, and age-1. When a mutation causes reduced activity of DAF-2, a receptor in the IIS, the lifespan more than doubles. The reduced IIS signaling results in less phosphorylation of DAF-16, which promotes DAF-16 migration to the nucleus. Once DAF-16 enters the nucleus, it contributes to stress response mechanisms and promotes the expression of genes that promote longevity [66]. SIR2 is a life-regulating and determining factor in *C. elegans*. It downregulates the IIS to extend lifespan, thereby promoting localization of DAF-16 to the nucleus and extending life [66,67]. Therefore, in this study, we investigated the effects of *H. cordata* ethyl acetate fraction on the expression of SIR-2.1 and DAF-2, as two important proteins involved in the longevity of *C. elegans*. Compared to the untreated group, the groups treated with 250 μg/mL and 500 μg/mL *H. cordata* ethyl acetate fraction showed 16.3% and 23.9% increases in SIR-2.1 protein expression, respectively (Figure 6C,D) and 11.4% and 23.4% decreases in DAF-2 protein expression, respectively (Figure 6E,F). Therefore, we determined that the ethyl acetate fraction of *H. cordata* helped to suppress aging in *C. elegans*. Moreover, as the dose of ethyl acetate fraction increased, we observed increased localization of DAF-16 in the nucleus (Figure 4C) and showed decreased DAF-2 protein content and increased SIR-2.1 protein content, suggesting that this fraction could affect aging in *C. elegans*.

### 3.9. LC/MS-MS and TLC Analysis

**Figure 7:**
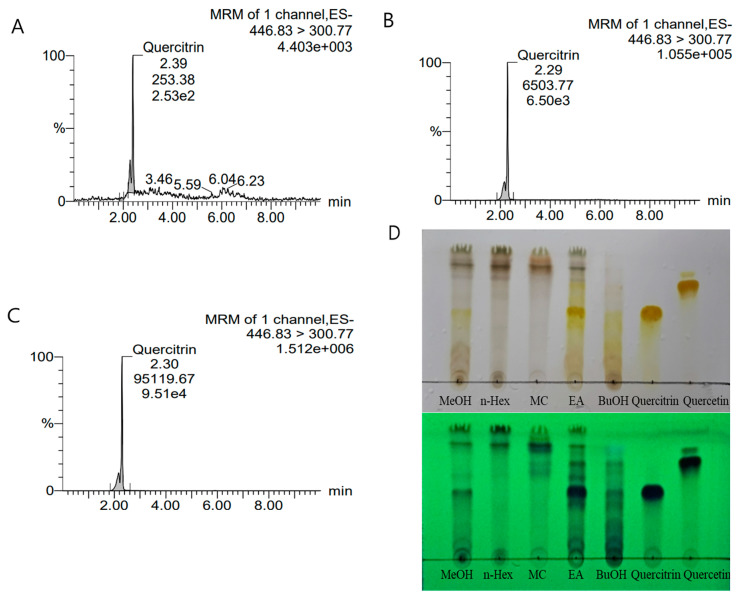
The LC/MSMS analysis results of *Houttuynia cordata* methanol extract and ethyl acetate fraction. Analysis results of standard quercitrin (**A**), quercitrin in the methanol extract (**B**), and quercitrin in the ethyl acetate fraction (**C**). *Houttuynia cordata* extracts and fractions were also analyzed using thin-layer chromatography (**D**). The upper part of (**D**) shows the TLC plate baked using 10% sulfuric acid, and the lower part shows the TLC plate viewed at 254 nm through UV light.

We performed LC/MSMS analysis to analyze and quantify the compounds with the highest concentrations in the methanol extract of *H. cordata* and in the ethyl acetate fraction, which was the fraction showing the strongest antioxidant and antiaging effects. The calibration curve equation was as follows:

y=205.825x+22.1797, r2=0.999

suggesting the reliability of the analysis results. The standards were diluted to concentrations of 1, 10, 100, and 1000 ppm; the peak retention time (RT) was 2.4, and the trace was 446.83 > 300.77. The area of each peak was calculated to represent the concentration of the given compound. Among the standards, quercitrin had the same RT and trace values; thus, the main constituent of the *H. cordata* extract and fraction was determined to be quercitrin. The concentration of quercitrin in 1000 ppm *H. cordata* extract was 31.5 ppb, and the concentration of quercitrin in the ethyl acetate fraction, obtained after polarity-based solvent fractionation, was 462.0 ppb, which was the highest concentration (Figure 7A–C and Table 4). Figure 7D also shows that the content of quercitrin is the highest in the EA fraction among all fractions and extracts. Since we added different concentrations of the ethyl acetate fraction used in all these analyses in the *C. elegans* experiments, and since the compound with the highest concentration in this fraction was quercitrin, we surmise that quercitrin potentially contributes to the antioxidant and antiaging effects in *C. elegans*.

## 4. Conclusions

In this study, we used *C. elegans* as a model to investigate the antioxidant and antiaging properties of *H. cordata*, a perennial herbaceous plant belonging to the family *Saururaceae*. The ethyl acetate fraction of *H. cordata* showed the best antioxidant activity and superior life-extending effects in *C. elegans* relative to those of the control group. Analysis of different concentrations of the ethyl acetate fraction (250 μg/mL, 500 μg/mL) revealed increased SOD and catalase activities and decreased ROS accumulation in *C. elegans*. The ethyl acetate fraction dose-dependently increased the expression of SOD-3::GFP in transgenic *C. elegans* strain CF1553 and significantly increased the maximum survival time under conditions of oxidative stress induced by high-concentration juglone. The *H. cordata* ethyl acetate fraction also dose-dependently reduced heat stress in *C. elegans*, thus increasing the survival rate. The ethyl acetate fraction also effectively decreased the expression of lipofuscin, an aging pigment produced during *C. elegans* aging and altered the expressions of DAF-2 and SIR-2.1, which are aging-related proteins. Thus, the ethyl acetate fraction of *H. cordata* could fundamentally help prevent aging and extend lifespan in *C. elegans*. LC/MSMS analysis identified a high concentration of quercitrin as the active constituent in the *H. cordata* ethyl acetate fraction. Thus, the antioxidant activity, extended lifespan, and suppressed aging in *C. elegans* could potentially be attributed to the quercitrin present in the *H. cordata* ethyl acetate fraction. Furthermore, the antioxidant and antiaging effects of quercitrin isolated and identified as a pure compound from the ethyl acetate fraction will be investigated using *C. elegans* to assess the influence of this component.

## Figures and Tables

**Table 1:** Total polyphenol and flavonoid contents of *Houttuynia cordata* Thunb methanol extract and fractions.

Extract and Fraction	Total Polyphenol (μg TAE/mg) ^(1)^	Total Flavonoid (μg QE/mg) ^(2)^
Methanol extract	161.9	346.4
*n*-Hexane fraction	171.0	286.2
Methylene chloride fraction	140.5	226.7
Ethyl acetate fraction	881.3	1941.1
*n*-Butanol fraction	249.1	670.6

^(1)^ Total polyphenol content analyzed as tannic acid equivalent (TAE) μg/mL of extract and fraction. ^(2)^ Total flavonoid content analyzed as quercetin acid equivalent (QE) μg/mL of extract and fraction.

**Table 2:** Effects of ethyl acetate fraction of *Houttuynia cordata* Thunb. on the oxidative and thermal stress tolerance of *C. elegans*.

Stress Condition		Mean Lifespan (Hours)	Maximum Lifespan (Hours)	Change in Mean Lifespan(%)	Log-Rank Test
1 mM Juglone	Control	13.5 ± 1.0	27	-	-
	250 μg/mL	23.3 ± 1.1	35	43.1	*** *p* < 0.001
	500 μg/mL	26.8 ± 1.2	40	57.0	*** *p* < 0.001
36 °C thermal tolerance	Control	14.3 ± 0.4	20	-	
	250 μg/mL	17.2 ± 0.5	24	20.0	*** *p* < 0.001
	500 μg/mL	18.9 ± 0.5	27	32.5	*** *p* < 0.001

Mean lifespan presented as mean ± standard error of mean (S.E.M) data. Change in mean lifespan compared with control group (%). The statistical significance of the difference between survival curves was determined by a log-rank test using the Kaplan–Meier survival analysis. Differences compared to the control were considered significant at *** *p* < 0.001 using one-way ANOVA.

**Table 3:** Effects of fractions from the *Houttuynia cordata* Thunb. on the lifespan of wild-type N2.

Fraction	Mean Lifespan (Day)	Maximum Lifespan (Day)	Change in Mean Lifespan	Log-Rank Test
Control	8.9 ± 0.2	16	-	-
Methanol	9.6 ± 0.2	17	7.3	* *p* < 0.05
*n*-Hexane	9.1 ± 0.2	17	-	-
Methylene chloride	9.6 ± 0.2	17	7.2	* *p* < 0.05
Ethyl acetate	10.9 ± 0.3	19	22.1	*** *p* < 0.001
*n*-Butanol	9.9 ± 0.3	19	11.2	** *p* < 0.01

Mean lifespan presented as mean ± S.E.M data. Change in mean lifespan was compared with the control group (%). The statistical significance of the difference between survival curves was determined by a log-rank test using the Kaplan–Meier survival analysis. Differences compared to the control were considered significant at * *p* < 0.05, ** *p* < 0.01, and *** *p* < 0.001.

**Table 4:** The LC/MSMS analysis results of *Houttuynia cordata* methanol extract and ethyl acetate fraction.

	Type	Concentration	Trace	RT	Response	ppb
Quercitrin	standard	1000 ppm	446.83 > 300.77	2.39	204,925.9	995.5
Methanol	sample			2.29	6503.775	31.5
Ethyl acetate	sample			2.30	95,119.67	462

## Data Availability

The original contributions presented in this study are included in the article. Further inquiries can be directed to the corresponding author.
